# Hand Ownership Is Altered in Teenagers with Unilateral Cerebral Palsy

**DOI:** 10.3390/jcm11164869

**Published:** 2022-08-19

**Authors:** Corinna N. Gerber, Didier L. Gasser, Christopher John Newman

**Affiliations:** Paediatric Neurology and Neurorehabilitation Unit, Department of Paediatrics, Lausanne University Hospital and Lausanne University, 1011 Lausanne, Switzerland

**Keywords:** cerebral palsy, hemiplegia, teenager, body ownership, rubber hand illusion, proprioceptive drift

## Abstract

We explored hand ownership in teenagers with unilateral cerebral palsy (UCP) compared with typically developing teenagers. Eighteen participants with UCP and 16 control teenagers participated. We used the rubber hand illusion to test hand ownership (HO). Both affected/non-affected hands (UCP) and dominant/non-dominant hands (controls) were tested during synchronous and asynchronous strokes. HO was assessed by measuring the proprioceptive drift toward the fake hand (as a percentage of arm length) and conducting a questionnaire on subjective HO. Both groups had significantly higher proprioceptive drift in the synchronous stroking condition for both hands. Teenagers with UCP showed a significantly higher proprioceptive drift when comparing their paretic hand (median 3.4% arm length) with the non-dominant hand of the controls (median 1.7% arm length). The questionnaires showed that synchronous versus asynchronous stroking generated a robust change in subjective HO in the control teenagers, but not in the teenagers with UCP. Teenagers with UCP have an altered sense of HO and a distorted subjective experience of HO that may arise from the early dysfunction of complex sensory–motor integration related to their brain lesions. HO may influence motor impairment and prove to be a target for early intervention.

## 1. Introduction

In children with unilateral cerebral palsy (UCP), the use of the affected hand in daily life, referred to as performance, is often below its use in a clinical environment, referred to as capacity [1,2]. This underuse is associated with developmental disregard [3] and is hypothesised to be due to the disproportional amount of attention required to use the affected limb in daily life [4]. Anecdotally, children with UCP report experiences that hint at potential issues with hand ownership (HO), described with phrases such as “I forget my hand” or “It’s as if my hand wasn’t there”. Appropriate HO is a prerequisite for the production of adapted voluntary movements [5]. Alterations in HO may impair affected hand use in UCP.

Body ownership relies on the spatial and temporal binding between perceptual events and is grounded in the brain’s ability to integrate multiple sources of sensory information [6]. The importance of visual–tactile integration in establishing HO is evidenced in the rubber hand illusion (RHI) [7], in which multisensory conflicts induce the self-attribution of a rubber hand. Viewing someone stroking a rubber hand (seen, not felt) while the viewer’s own hand, which is occluded from view, is being stroked synchronously (felt, not seen) induces a sense of ownership for the rubber hand, with the illusion of feeling the strokes being applied to it [8]. The viewer’s own hand is perceived in a position displaced towards the fake hand, a phenomenon named proprioceptive drift (PD). Illusory ownership and PD decrease or disappear when strokes are applied asynchronously to the rubber and real hands [9].

Illusory ownership can be induced in healthy adults [7] and typically developing (TD) children. Applying the RHI in children demonstrated that the multisensory processes underlying body representations are different in children aged 4 to 9 years compared with adults [10]. For HO, these younger children rely more strongly on the sight of their hands and less on their proprioception than adults. A further study in 10- to 13-year-olds [11] showed that pointing responses reached adult levels at 10 to 11 years, showing that, from this age, children integrate their hands by using an adult-like balance of sensory cues.

Adults with unilateral stroke seem to have a looser sense of HO and experience stronger illusory effects on the affected hand [12]. The effects of early brain anomalies on the development of HO are unknown. It is likely that the early damage to the motor and sensory cortex and pathways typically encountered in UCP disrupts the development of HO, which is important for both hand integration and motor function; however, to this date, body ownership has not been explored in cerebral palsy. In this study, we aimed to explore HO in teenagers with UCP compared with TD controls, questioning whether hand ownership is altered in UCP. We hypothesised that RHI could be induced in both groups, and that the RHI would have stronger effects on the affected hand in youngsters with UCP.

## 2. Materials and Methods

### 2.1. Study Design and Setting

This pilot case–control study was approved by the regional ethics committee (CER-VD decision 2017-0208) and was conducted between July 2018 and July 2019 at Lausanne University Hospital (CHUV), Switzerland.

### 2.2. Participants

We aimed to include a minimal convenience sample of 15 participants in each group (UCP and TD), in line with previous studies on the RHI [13]. Youngsters aged 10–20 years old were included in the study, based on the previous study on TD children who were shown to reach an adult perception of the bodily self by 10 years [11].

Participants with UCP were recruited from the paediatric neurorehabilitation clinic of Lausanne University Hospital. The inclusion criteria were: (i) UCP diagnosed by a paediatric neurologist, (ii) age 10 to 20 years, (iii) hand use classified as Manual Ability Classification System (MACS) level I-III, and (iv) ability to hold each hand, palm down, on a table.

We recruited controls among the acquaintances of the study participants and children of Lausanne University Hospital’s paediatric department’s collaborators. The inclusion criteria for TD controls were: (i) age 10 to 20 years, (ii) no known history of brain lesions or disorders, and (iii) no known sensory and/or motor impairment of the upper extremities.

The exclusion criteria for all participants were: (i) surgery at the trunk or upper limb level within the last six months before inclusion in the study; (ii) botulinum toxin injections in the upper limbs within the three months before inclusion in the study; (iii) any clinically significant disease (e.g., renal failure, hepatic dysfunction, and cardiovascular disease); (iv) known or suspected non-compliance; (v) inability to follow the procedures of the study, for example, due to language problems or behavioural issues; (vi) cognitive age estimated below 10 years; and (vii) severe visual impairments, including hemianopsia.

The TD controls had one test session during which the RHI experiment was conducted, and participants with UCP had an additional session to assess secondary outcomes. Written informed consent was obtained from participants older than 14 years and from their legal guardians if they were younger than 18 years.

### 2.3. Procedure

**Figure 1:**
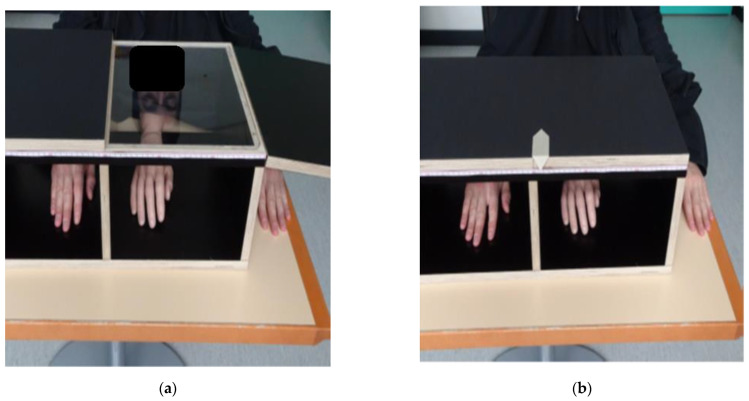
Rubber hand illusion experimental setup. (**a**) Stimulation phase: during the stroking with a paintbrush, the participant sees a rubber hand while their real hand (here, the right hand) is hidden. (**b**) Measurement condition. Both the real and the rubber hands are hidden. The participant indicates where he feels the index finger of his (in this case, right) hand by instructing the experimenter to move the cursor to the right or to the left.

The RHI experiment followed the protocol established by Cowie et al. [11]. We used distances relative to the participant’s arm length for the setup (Figure 1) and the measurement of PD. To account for different hand appearances, we selected, from three pairs of fake hands (silicon prosthetics), the one most resembling the participant’s hand: adult female, adult male, and child (Ortho Kern SA, Lausanne, Switzerland).

To induce the RHI illusion, the experimenter stroked the visible fake hand and the hidden real hand of the participant with two identical paint brushes in two different stroking conditions (synchronously and asynchronously).

The affected hand (UCP) or the non-dominant hand (TD) was tested first, and the two stroking conditions were applied randomly. For each side and condition, the experiment consisted of four baseline measures with the fake and real hands hidden in the box, followed by a stimulation phase (2 min) and four post-stimulation phases (each 20 s), where the fake hand was visible while the real hand was hidden in the box. After each baseline, stimulation, and post-stimulation phase, both hands were hidden for the PD measurement.

### 2.4. Outcome Measures

We measured PD as a primary outcome to quantify the RHI. To avoid visual cues (e.g., the midline of the box), a black sheet of paper was placed over it before each measurement. The experimenter then moved a cursor (Figure 1) along the box following the participant’s instruction of “left” or “right” until the participant felt confident that the ruler indicated the position of their index finger. The distance of the indicated position from the midline was measured in millimetres.

The PD was normalised to arm length and calculated as follows:

$$PD=\frac{\left(\frac{\sum BL_{i}}{4}\right)-\frac{Stim\;+\;\sum Post_{i}}{5}}{L}*100$$

in which *BL* is the baseline measure, *Stim* is the stimulation phase, *Post* is the post-stimulation phase, *L* is the arm length of the participant, and *i* is the number of measurements.

The subjective sense of ownership of the rubber hand was assessed as a secondary outcome with a 6-statement questionnaire adapted from Botvinick and Cohen [9] and Burin et al. [12]. Three statements explored the predicted phenomena: (i) I felt the touch of the paintbrush where I saw the rubber hand was touched; (ii) It seemed as if the touch I felt was caused by the paintbrush touching the rubber hand; and (iii) It felt as if the rubber hand was my hand. The three control statements with no expected effect were: (i) I felt my hand drifting towards the rubber hand; (ii) It seemed as if the touch I was feeling came from somewhere between my own hand and the rubber hand; (iii) It felt as if my real hand was turning rubbery. Participants responded on a 7-point Likert scale with the following coding: no, definitely not (−3); no (−2); no, not really (−1); neither yes nor no (0); yes, a little (1); yes, a lot (2); and yes, really a lot (3).

We measured the motor function and capacity, as well as sensory functioning, of participants with UCP as potential co-factors of HO. For this purpose, we used the second version of the Melbourne Assessment of Unilateral Upper Limb Function (MA2), the Assisting Hand Assessment (AHA), 2-point discrimination, and a joint-position sense test.

The MA2 and AHA were both video recorded with video-based scorings. The MA2 is a valid and reliable measure of upper limb function in children with central motor disorders [14]. Sub-scores on a scale from 0–100 are provided for the range of motion, dexterity, fluency, and accuracy. The AHA measures the spontaneous use of the more-affected hand during bimanual activities. The 22 items describe actions under the following sub-headings: general use, arm use, grasp and release, fine motor adjustments, coordination, and pace. We used two versions of the AHA: the kids-AHA [15] for participants under 12 years old, and the Ad-AHA [16] for adolescents aged 13 years or older.

We measured sensory function according to the protocol described by Cooper et al. [17]. Static 2-point discrimination was measured with an aesthesiometer (Baseline, Fabrication Enterprise Inc., White Plains, NY, USA) on the palmar side of the distal phalanx of both index fingers with pressure to the point of skin blanching. The minimal distance of the two pressure points that the patient actually perceived (with eyes closed) as two distinct points was recorded. To measure joint position sense, a component of proprioception, the tester fixed the proximal and middle phalanges, held the patient’s distal phalanges of the index finger, and moved the latter up and down passively. The patient had to detect the direction of movement (up/down) with their eyes closed, and the correct answers out of five trials were recorded.

### 2.5. Statistical Analyses

Due to the small sample size, non-parametric tests were used. We compared the synchronous and asynchronous stroking conditions using the Wilcoxon Signed Ranks Test for both hands of both groups separately. Subsequently, we performed a Mann–Whitney test to compare the PD of the participants with UCP’s affected hand with the controls’ non-dominant hand and of the participants with UCP’s non-affected hand with the controls’ dominant hand for the synchronous stroking condition.

Spearman correlations were calculated to investigate associations between the difference in perceived hand position (i.e., PD) after the synchronous stroking of the affected hand with clinical co-factors.

Statistical analyses were performed with SPSS 26 (IBM, Armonk, NY, USA). Alpha was set at 0.05, and Bonferroni corrections were applied where necessary.

## 3. Results

Eighteen participants with UCP (mean age: 13 y 10 m; SD 2 y 10 m, six females) and sixteen TD controls (age 14 y 1 m; SD: 3 y 0 m, 6 females) were included. Half (nine) of the participants with UCP had right-sided hemiparesis, half had left-sided hemiparesis, and nine participants were at MACS level I, five at MACS level II, and four at MACS level III. None had previously participated in an RHI study.

**Figure 2:**
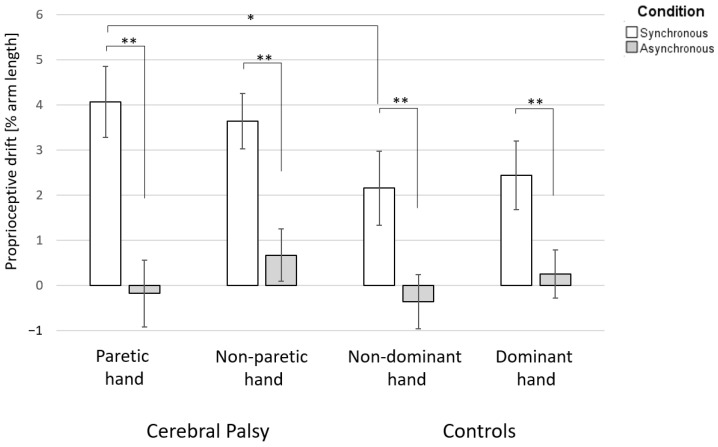
Proprioceptive drift in teenagers with unilateral cerebral palsy and typically developing teenagers. A positive value of proprioceptive drift indicates a drift of the felt index finger location towards the rubber hand, while a negative value shows a drift away from the rubber hand. Significant differences are indicated as follows: * *p* < 0.05, and ** *p* < 0.01.

The difference in PD between the synchronous and asynchronous conditions was significant in both groups and for both hands (Figure 2).

For group analyses of the PD after the synchronous stroking condition, we found a significant difference between the affected hand of participants with UCP and the non-dominant hand of TD participants (median PD of affected hands: 3.4% arm length; median PD of non-dominant hands: 1.7% arm length; the distributions in the two groups differed significantly, Mann–Whitney U = 201, n1 = 18, n2 = 16, *p* = 0.049 two-tailed). However, no difference was observed between the unaffected and dominant hands (median PD of unaffected hands: 3.5% arm length; median PD of dominant hands: 2.0% arm length; the distributions in the two groups differed significantly, Mann–Whitney U = 189, n1 = 18, n2 = 16, *p* = 0.121, two-tailed).

**Figure 3:**
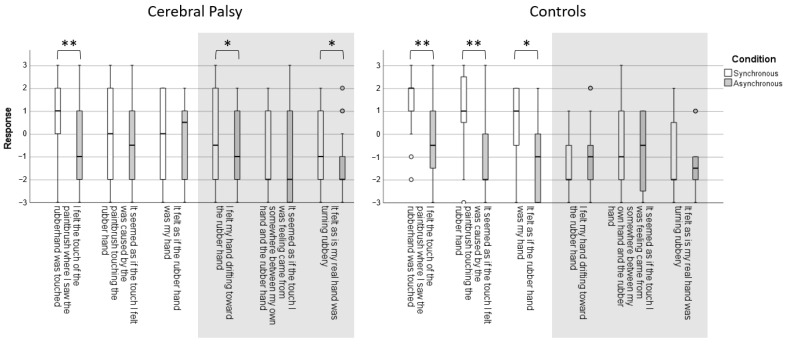
Subjective ratings of hand ownership in teenagers with unilateral cerebral palsy and typically developing teenagers. Responses to statements on subjective body ownership were given on a 7-point Likert scale ranging from “do completely agree” +3 to “do not agree at all” −3, where 0 corresponded to neither agreeing nor disagreeing. Greyed areas indicate control statements. Significant differences are indicated as follows: * *p* < 0.05, and ** *p* < 0.01.

The HO questions showed that the controls had a significantly higher subjective experience of HO over the fake hand after the synchronous stroking condition of their non-dominant hand (3/3 statements), whereas no effect was found for the control questions (0/3 statements). In children with CP, the results for the subjective experience of HO were inconsistent for the questions regarding their affected hand (Figure 3).

The results of the clinical co-factors are displayed in Table 1. There were no significant correlations between the clinical co-factors and PD.

## 4. Discussion

Teenagers with UCP were responsive to the RHI, whereas, for TD teenagers, the RHI effects were dependent on the synchronicity of the stroking. The effects of the RHI on teenagers with UCP demonstrated that they had an altered sense of ownership of their paretic hands. The RHI experiment generated a significantly higher PD of their paretic hand towards the rubber hand than for the non-dominant hand in TD controls. Synchronous versus asynchronous stroking generated a robust change in subjective hand ownership in TD teenagers, but not in teenagers with UCP.

In TD children, the perceived hand position, which requires implicit hand ownership, seems to mature during late childhood. In a study that applied the RHI to children, Cowie et al. [10] showed that, during the first decade, children had consistently higher levels of PD than during the second decade, when PD approached adult levels. This was in contrast to the explicit hand ownership (the phenomenological experience in which the ownership of the hand is experienced consciously) that was measured through questions on the subjective experience of ownership, which is present from early childhood and demonstrates no significant development between 4 years of age and adulthood. This developmental trajectory of hand ownership was attributed to an early reliance on visual–tactile integration to develop a perception of bodily layout and propriety, followed by later developing visual–proprioceptive processes to consolidate the perception and ownership of body parts [10].

In teenagers with UCP, both processes seem to be disrupted. The persistence of a significantly higher PD for the paretic hand throughout the teenage years into adulthood most likely demonstrates a prolonged reliance on visual input to contribute to hand ownership, with a potential failure in the expected maturation of visual–proprioceptive processes. In contrast, subjective ownership was distorted in teenagers with UCP compared with TD teenagers, with an atypical pattern of responses to the questionnaire. Among the statements that were devised to detect illusory ownership, there was a clear dissociation between the positive response to the sensory illusion (“I felt the touch of the paintbrush where I saw the rubber hand was touched”) and the negative response to the ownership illusion (“It felt as if the rubber hand was my hand”). This contrasts with findings in adults after stroke, who experience a change in the belief of ownership over the rubber hand [12], pointing towards a specific influence of early injury in the maldevelopment of subjective body ownership. For the statements with no expected effect, teenagers with UCP were more likely to report that they felt their hand drifting towards the rubber hand, and that they felt their real hand turning rubbery.

The complex construct of subjective hand and bodily ownership relies on the interplay of brain areas, including the fronto-parietal network and temporo-parietal junction, as demonstrated by functional MRI [18,19,20,21] and transcranial magnetic stimulation [22] experiments conducted during the RHI. Early anomalies in frontal, fronto-parietal, or larger brain areas, which are implicated in sensory–motor processing and are classically affected in cerebral palsy, may limit these children’s ability to develop a typical sense of body ownership for their affected body parts, as demonstrated in our findings regarding the paretic hand of teenagers with UCP.

The effects of atypical HO on the development of hand motor skills in children with UCP and, conversely, the effects of manual motor impairment on the development of HO remain to be determined. We were unable to demonstrate any association between impairment in HO, as measured by PD, and measures of motor or sensory function. Voluntary movements have been suggested as important in supporting proper multisensory integration and, therefore, for subjective body ownership [12,23]. In adults with paresis, the decrease in the number of movement-related signals, which disrupts the normal integration of afferent and efferent signals for the arm, weakens body ownership. However, there is also evidence that atypical body ownership can lead to motor difficulties. A small case series of adults with right brain damage and hemiplegia, affected by an atypical form of hemisomatoagnosia, revealed that the pathological self-attribution of an alien hand directly affected the patients’ motor programme by modifying motor awareness, sense of agency, and action execution [24]. Hence, distorted HO due to early brain damage might hinder optimal motor development and performance in people with CP.

Could HO become a target for therapy? Mirror therapy is an approach based on the manipulation of hand ownership [25]. In mirror therapy, patients look at the reflection of their non-impaired upper limbs on a mirror placed at their midlines while they perform symmetrical upper-limb movements. The reflection generates the multisensory illusion that the paretic hand is functioning normally through a self-attribution of the reflection in the mirror, that is, the mirror illusion. Interestingly, mirror therapy has failed to exhibit the same efficacy in children with UCP [25] compared with adults after stroke [26]. While adults have a life of experienced HO before their stroke that may underpin the response to mirror therapy, it is possible that children with UCP who have developed from early life with atypical HO may not be as responsive to the mirror illusion and therapy. This hypothesis is supported by recent research suggesting that cross-modal visuo-tactile integration is dependent on body ownership [27]. Therefore, the effectiveness of cross-modal rehabilitative interventions might depend on the integrity of the patients’ body ownership. The process of body ownership develops from infancy [28] and has already been strongly established based on visual–tactile integration by early childhood [10]. Therefore, early sensorimotor intervention, specifically geared at supporting multisensory integration as related to hand movement and agency, may support a more typical development of HO, thus supporting future hand function. Such an intervention could focus on driving the child’s visual, tactile, and proprioceptive attention towards the affected hand, for example, by the activation of luminous and vibratory cues synchronised with guided or voluntary hand movement.

Our sample size may have underpowered our detection of potential differences between groups. Specifically, Figure 2 hints that PD for the non-paretic hand of teenagers with UCP may be higher than that for the dominant hand of TD teenagers. Despite not attaining statistical significance, this could be a sign of a more diffuse issue in the development of body ownership beyond the paretic body parts. Our sample size could have restricted our ability to demonstrate meaningful associations between body ownership and motor or sensory functions. Studies with larger sample sizes, a prospective observation of the development of HO throughout childhood, and the integration of additional co-factors, including brain imaging, are necessary to further define the extent and likely variability of HO issues in UCP, as well as their neurobiological underpinnings.

In conclusion, teenagers with UCP have an altered sense of HO with an excessive reliance on visual input, evocative of an abnormal maturation of visual–proprioceptive processes, and a distorted subjective and explicit experience of HO that most probably stems from the early dysfunction of complex sensory–motor integration related to their brain lesions. Abnormal HO most likely interacts with motor impairment and may prove a target for early sensorimotor intervention to improve the development of HO and, ultimately, hand function.

## Figures and Tables

**Table 1:** Clinical measures of motor and sensory function and correlations with proprioceptive drift of the affected hand in teenagers with unilateral cerebral palsy.

			Melbourne Assessment 2					
		AHA_Score (%)	RoM (%)	Precision (%)	Dexterity (%)	Fluidity (%)	TPD (mm)	JPS (N)
**Proprioceptive Drift** (synchronous stroking)	**Mean (SD)**	69.7 (22.0)	77.6 (23.5)	90.4 (16.2)	71.6 (24.2)	71.9 (25.5)	1.5 (2.6)	4.3 (0.8)
	**Correlation Coefficient**	−0.011	−0.227	−0.339	−0.094	−0.142	−0.007	−0.236
	***p***	0.964	0.364	0.169	0.712	0.573	0.978	0.345
	**N**	18	18	18	18	18	16	18

Abbreviations: AHA, Assisting hand assessment; RoM, Range of motion; TPD, Two-point discrimination; JPS, joint position sense; *p*, *p*-value; N, number.

## Data Availability

The data that support the findings of this study are available upon request from the corresponding author. The data are not publicly available due to privacy or ethical restrictions.
